# Supraphysiological glutamine as a means of depleting intracellular amino acids to enhance pancreatic cancer chemosensitivity

**DOI:** 10.21203/rs.3.rs-3647514/v1

**Published:** 2023-11-30

**Authors:** Hayato Muranaka, Sandrine Billet, Carlos Cruz-Hernández, Johanna ten Hoeve, Gabrielle Gonzales, Omer Elmadbouh, Le Zhang, Bethany Smith, Mourad Tighiouart, Sungyong You, Mouad Edderkaoui, Andrew Hendifar, Stephen Pandol, Jun Gong, Neil Bhowmick

**Affiliations:** Cedars-Sinai Medical Center; Cedars-Sinai Medical Center; Cedars-Sinai Medical Center; University of California Los Angeles; Cedars-Sinai Medical Center; Cedars-Sinai Medical Center; Cedars-Sinai Medical Center; Cedars-Sinai Medical Center; Cedars Sinai Medical Center; Cedars-Sinai Medical Center; Cedars-Sinai Medical Center; Cedars-Sinai Medical Center; Cedars-Sinai Medical Center; Cedars-Sinai Medical Center; Cedars-Sinai Medical Center

## Abstract

Limited efficacy of systemic therapy for pancreatic ductal adenocarcinoma (PDAC) patients contributes to high mortality. Cancer cells develop strategies to secure nutrients in nutrient-deprived conditions and chemotherapy treatment. Despite the dependency of PDAC on glutamine (Gln) for growth and survival, strategies designed to suppress Gln metabolism have limited effects. Here, we demonstrated that supraphysiological concentrations of glutamine (SPG) could produce paradoxical responses leading to tumor growth inhibition alone and in combination with chemotherapy. Integrated metabolic and transcriptomic analysis revealed that the growth inhibitory effect of SPG was the result of a decrease in intracellular amino acid and nucleotide pools. Mechanistically, disruption of the sodium gradient, plasma membrane depolarization, and competitive inhibition of amino acid transport mediated amino acid deprivation. Among standard chemotherapies given to PDAC patients, gemcitabine treatment resulted in a significant enrichment of amino acid and nucleoside pools, exposing a metabolic vulnerability to SPG-induced metabolic alterations. Further analysis highlighted a superior anticancer effect of D-glutamine, a non-metabolizable enantiomer of the L-glutamine, by suppressing both amino acid uptake and glutaminolysis, in gemcitabine-treated preclinical models with no apparent toxicity. Our study suggests supraphysiological glutamine could be a means of inhibiting amino acid uptake and nucleotide biosynthesis, potentiating gemcitabine sensitivity in PDAC.

## Introduction

Pancreatic cancer has one of the highest mortalities of all cancers, in part due to the rapid development of resistance to systemic therapy [1, 2]. Several efforts have been directed at exploiting metabolic vulnerabilities that mediate and promote therapeutic resistance. Glutamine (Gln) is considered an essential amino acid for growth and survival in pancreatic ductal adenocarcinoma (PDAC) [3, 4], and disruption of Gln metabolism or Gln deprivation are reported to sensitize PDAC cells to chemotherapy, such as gemcitabine [5, 6]. However, despite some promising early effects on proliferation, loss of therapeutic effects of Gln restriction has been observed [7]. Additionally, such strategies were likely not effective, since PDAC tumors inherently have low glutamine levels compared to benign adjacent tissues [8] and scavenge extracellular proteins by oncogenic KRAS-driven macropinocytosis to adapt to nutrient restricted microenvironments [9, 10]. Furthermore, cancer cells reprogram their metabolism to utilize other carbon sources for survival, such as asparagine and aspartate [11–13]. Therefore, alternative approaches to target Gln metabolism in PDAC are required.

There is growing evidence to support the use of Gln supplementation to reduce gastrointestinal symptoms associated with chemotherapy [14, 15]. However, Gln diet supplementation in cancer patients is controversial, since there is a clear possibility of supporting tumor cell growth. There are studies demonstrating Gln supplementation could inhibit melanoma tumor growth and sensitized tumors to targeted therapy via epigenetic reprogramming [16, 17]. High dose Gln was approved by the FDA for patients with sickle cell disease [18–20]. However, there is still limited understanding of the effects of supraphysiological Gln (SPG) concentrations on cancer. This is at least partially due to the difficulties of manipulating and monitoring Gln concentrations *in vivo*, especially in the tissues [21]. Interestingly, recent study demonstrated directly enhancing intratumoral Gln abundance affects anti-tumor immunity in subcutaneous tumor xenograft in immunocompetent mice [22]. In oocytes expressing human alanine serine cysteine transporter 2 (ASCT2), the primary Gln transporter in cancer cells, an addition of SPG concentrations inhibited uptake of neutral amino acids [23]. Since inhibition of amino acid availability generally results in cancer cell growth inhibition, we hypothesized that SPG could suppress cancer cell growth via inhibition of amino acid uptake in cancer cells. Moreover, there is an interesting precedent where suppressing androgen receptor signaling is the primary intervention for advanced prostate cancer, yet supraphysiological androgen administration can produce paradoxical responses of tumor growth inhibition [24]. These observations motivated the testing SPG for the treatment of PDAC.

Pancreatic cancer cells exhibit complex metabolic reprogramming including elevated glucose, amino acid, and lipid metabolism, in part due to mutations in K-Ras and TP53. Long-term chemotherapy such as gemcitabine treatment can also induce elevated aerobic glycolysis and pyrimidine biosynthesis as well as reprogramming of lipid metabolism [25, 26]. Glutamine can exist in either of two enantiomeric forms, L-glutamine (L-Gln) and D-glutamine (D-Gln). L-Gln, but not D-Gln, can be metabolized. In the current study, we demonstrated the efficacy of supraphysiological concentrations of both L-Gln and D-Gln, that induced paradoxical cell growth inhibition and gemcitabine sensitivity through the suppression of amino acid uptake. Among three commonly prescribed chemotherapies for PDAC patients, gemcitabine-treated cells demonstrated significant amino acid dependencies and was found to be especially sensitive to SPG^L^- and SPG^D^-induced amino acid deprivation. The findings support Gln supplementation as a viable therapeutic strategy in PDAC patients treated with gemcitabine.

## Materials and Methods

All reagents, cell culture, transcriptional, metabolic, and animal studies as well statistical analysis are described in detail Supplementary Materials and Methods.

## Results

### Supraphysiological glutamine concentrations inhibit cell growth in PDAC cells

**Figure 1:**
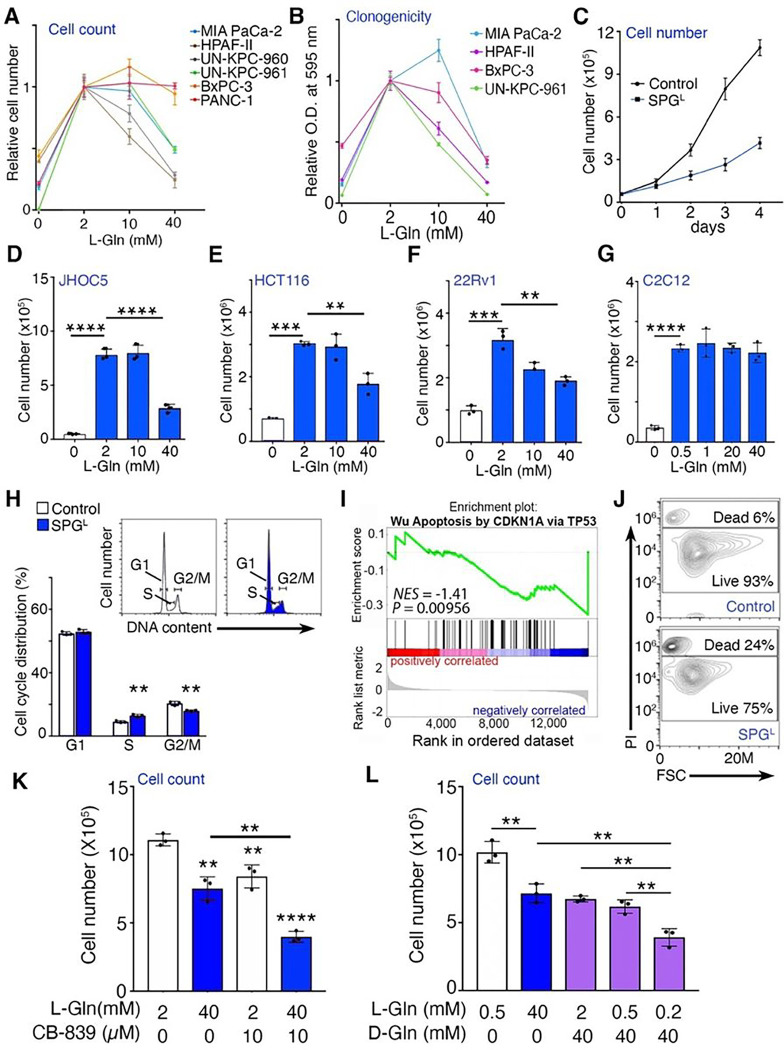
Supraphysiological Gln inhibits cancer cell growth. (**A**) Effects of different L-Gln concentrations on proliferation of PDAC cell lines. Cells were cultured in indicated L-Gln concentrations for four days and live cells were counted (n = 3). (**B**) Effects of different L-Gln concentrations on clonogenicity of PDAC cell lines. Cells were cultured in indicated L-Gln concentrations for 9–12 days and clonogenicity was measured by Crystal Violet staining (n=3). (**C**) Cell number of MIA PaCa-2 cells incubated with 2 mM (control) or 40 mM L-Gln (SPG^L^) were sequentially counted (n = 3). (**D-G**) Cell proliferation of human ovarian, colon, and prostate cancer cell lines (JHOC5, HCT116, 22Rv1, respectively) and mouse myoblast cell line (C2C12) following culturing in indicated concentrations of L-Gln for four days (n = 3). (**H**) Cell cycle distribution analysis of propidium iodide-stained MIA PaCa-2 cells (live cells) following indicated treatments for four days (n = 3). (**I**) GSEA of RNA-seq data indicated the enrichment of apoptosis related gene sets in SPG^L^ - treated MIA PaCa-2 cells compared to control. MIA PaCa-2 cells were incubated with SPG^L^ or control for 48 hrs (n = 3). (**J**) Cell death of MIA PaCa-2 cells cultured in control or SPG^L^ for four days, measured by propidium iodide uptake. (**K**) Cell proliferation of MIA PaCa-2 cells following incubation with CB-839 and L-Gln for four days (n = 3). (**L**) Cell proliferation of MIA PaCa-2 cells cultured with in indicated concentrations of L-Gln and D-Gln for four days (n = 3). Values are mean ± SEM; *P < 0.05, **P < 0.01, ***P < 0.001, ****P < 0.0001.

Gln is the most extensively studied amino acid in cancers including PDAC. Yet the effects of Gln supplementation in PDAC has not been fully evaluated. For better understanding of the relationship between glutamine concentrations and cell growth, we cultured PDAC cell lines with different concentrations of Gln including supraphysiological concentrations and examined the cell proliferation and clonogenicity. Human plasma Gln concentrations vary between 400 and 600 μM and tissue concentrations between 2 and 20 mM intracellular water [27], thus, we used 0–40 mM Gln in the experiments to cover physiological – supraphysiological Gln concentrations. Of note, there was no significant change in clonogenicity with 0.2 to 20 mM L-Gln, while 0, 30, and 40 mM L-Gln concentrations significantly suppressed PDAC cell growth (Supplementary Fig. S1A). As shown in the previous studies, L-Gln deficient media significantly decreased cell growth in six PDAC lines that we tested, compared to the standard media containing 2 mM L-Gln (Fig. 1A) [3, 4]. Interestingly, supraphysiological glutamine concentrations (SPG^L^: 10–40 mM L-Gln) had differential effects on cultured PDAC cell lines. The human PDAC cell line HPAF-II and murine UN-KPC-960 cells were especially sensitive to SPG^L^, while human MIA PaCa-2 and murine UN-KPC-961 cells were slightly less sensitive. The cell proliferation of human PANC-1 and BxPC-3 cells did not seem to be affected by SPG^L^ exposure for 4 days. However, under 10 days of incubation, the diverse PDAC lines had remarkable responses to both 0 mM and 40 mM L-Gln, where colony formation was significantly suppressed compared to ones cultured with media containing standard 2 mM L-Gln (Fig. 1B). A time course analysis of cell growth in MIA PaCa-2 cells with SPG^L^ suggested a significant growth inhibition by SPG^L^ at day 2–4 (Fig. 1C). The growth inhibitory effect of SPG^L^ seen in PDAC cell lines were also observed in human ovarian (JHOC5), colon (HCT116), and prostate (22Rv1) cancer cell lines (Fig. 1D–F). However, cell growth of the benign murine C2C12 myoblasts were not affected by SPG^L^ (Fig. 1G). Collectively, these results suggest the supraphysiological concentrations of glutamine can produce paradoxical responses leading to tumor growth inhibition. Cell cycle analysis of propidium iodide-stained MIA PaCa-2 cells identified that SPG^L^ caused an increase in S phase and decrease in G2/M phase, compared to control (Fig. 1H). In accordance with this finding, RNA sequencing analysis revealed the change in histone gene expression predominantly associated with S phase (*H2BC12, H2BC21* and *H2BC5*) by SPG^L^ (Supplementary Fig. S1B). Additionally, Gene Set Enrichment Analysis (GSEA) of the same data set suggested that SPG^L^ induced apoptotic programming, corroborated by flow cytometric analysis of PI-positive dead cells (Fig. 1I, J). Interestingly, while Gln deprivation induced an activation of Gln-responsive genes including the Mitogen-activated protein kinase (MAPK) pathway, the unfolded protein response (UPR) and endoplasmic reticulum (ER) stress, serine metabolism, and Gln metabolism, SPG did not cause similar effects (Supplementary Fig. S1C). Together, these data suggest that SPG^L^ promoted S phase cell cycle arrest and increased cell death while it has distinct impact on gene expression compared to Gln deprivation.

Since L-Gln can fuel cancer cells through anaplerosis, we tested if the anticancer effects of SPG^L^ can be further potentiated by simultaneously blocking glutamine metabolism. We found that the potent glutaminase inhibitor, CB-839, in the context of supraphysiological L-Gln treatment, significantly limited MIA PaCa-2 cell growth compared to either treatment alone (Fig. 1K). This result led us to further examine if supraphysiological D-Gln elicits greater inhibitory effects than supraphysiological L-Gln. In the presence of 2 mM L-Gln, D-Gln (40 mM, SPG^D^) inhibited cell growth of MIA PaCa-2 cells, like 40 mM L-Gln alone (Fig. 1L). However, under low L-Gln condition (0.2 mM) reflecting the Gln concentrations in non-peripheral regions (close to tumor core) of PDAC tumors, SPG^D^ treatment achieved lower cell counts over SPG^L^ (*P* < 0.01) [28]. Furthermore, the clonogenicity were decreased by SPG^D^ under physiologic concentrations of L-Gln in MIA PaCa-2, BxPC-3, and UN-KPC-961 cells (0.5 mM; Supplementary Fig. S1D-F). These results indicated the more effective growth inhibition by SPG^D^ compared to SPG^L^ in low Gln condition.

### Supraphysiological glutamine concentrations affect intracellular amino acid levels

**Figure 2:**
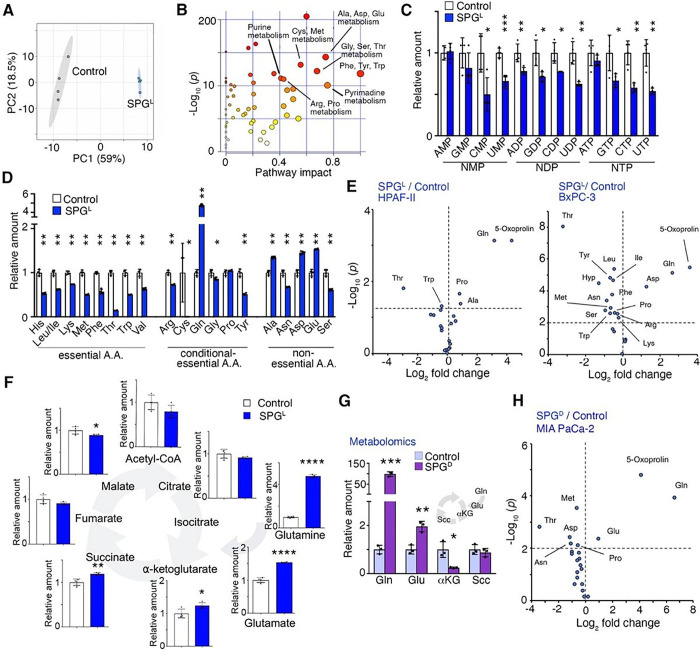
Supraphysiological Gln affects intracellular nucleotide and amino acid levels in PDAC. (**A**) Principal component analysis (PCA) score plot of metabolites from MIA PaCa-2 cells cultured in 2 mM L-Gln (control) or 40 mM L-Gln (SPG^L^) for 48 hrs (n = 4). (**B**) Pathway impact analysis of metabolites from MIA PaCa-2 cells cultured in control or SPG^L^. The color and size of each circle is based on p-values (yellow: higher *p*-values and red: lower *p*-values) and pathway impact values (the larger the circle the higher the impact score) calculated from the topological analysis, respectively (n = 4). (**C, D**) Intracellular nucleotide and amino acid levels were measured in control and SPG^L^-treated MIA PaCa-2 cells (n = 4). (**E**) Volcano plots of differentially enriched amino acids in HPAF-II and BxPC-3 cells in response to SPG^L^ treatment (HPAF-II, *P* < 0.05; BxPC-3, *P* < 0.01; n = 3). (**F**) The effect of SPG^L^ on TCA cycle intermediates in MIA PaCa-2 cells (n = 4). (**G**) Glutaminolysis and TCA cycle intermediates in MIA PaCa-2 cells cultured in 0 (control) or 40 mM D-Gln (SPG^D^) in the presence of 0.5 mM L-Gln (n = 4). (**H**) Volcano plot depicted differentially enriched amino acids in MIA PaCa-2 cells between the SPG^D^ treatment and control (*P* < 0.01, n = 3). For metabolomics experiments in A-H, samples were collected from the cells cultured with indicated treatment for 48 hrs. Values are mean ± SEM; *P < 0.05, **P < 0.01, ***P < 0.001, ****P < 0.0001.

We next examined the mechanisms leading to the paradoxical growth inhibitory effects of supraphysiological L-Gln and D-Gln on PDAC cells. Metabolomic analysis of MIA PaCa-2 cells was performed after the incubation with SPG^L^ for 48 hrs. Of note, at the 48 hrs time-point which was earlier than the time-point for proliferation assay (four days) or clonogenicity assay (9–12 days), there were very few dead cells in SPG^L^ -treated dishes. All data was normalized to live cell number. Additionally, floating dead cells were discarded in the metabolomics assays. Principal component analysis (PCA) demonstrated clear metabolomic difference between SPG^L^ and control (2 mM L-Gln) treated MIA PaCa-2 cells (Fig. 2A). A pathway impact plot indicated ‘phenylalanine, tyrosine, and tryptophan biosynthesis,’ ‘pyrimidine metabolism,’ ‘alanine, aspartate/glutamate metabolism,’ ‘glycine, serine/threonine metabolism,’ and ‘arginine biosynthesis’ as the most significantly affected networks by SPG^L^ treatment (Pathway Impact > 0.6, Fig. 2B). As expected, SPG^L^ elevated mitochondrial respiration in MIA PaCa-2 cells (Supplementary Fig. S2A). Closer examination delineated the unexpected enrichment of nucleosides at the cost of depleted nucleotides by SPG (Fig. 2C, Supplementary Fig. S2B). Importantly, essential amino acids and conditionally essential amino acids (except Gln and proline (Pro)) were significantly reduced by SPG^L^ (Fig. 2D). Among non-essential amino acids, alanine (Ala), aspartate (Asp) and glutamate (Glu) (known Gln metabolites) were elevated, while asparagine (Asn) and serine (Ser) were decreased. Notably, intracellular amino acid levels in HPAF-II and BxPC-3 cells were also diminished by SPG^L^ (Fig. 2E). While there was an increase in glutaminolysis and TCA cycle intermediates by SPG^L^, it was not observed in SPG^D^-treated cells (Fig. 2F, 2G). Similar to SPG^L^, SPG^D^ (in the presence of 0.5 mM L-Gln) reduced amino acid enrichment in MIA PaCa-2 cells (Fig. 2H). These results indicate that both SPG^L^ and SPG^D^ have an ability to reduce intracellular amino acids, especially essential amino acids, while SPG^D^ has an additional effect on glutaminolysis and TCA cycle, that may contribute to their growth inhibitory effects in PDAC cells.

**Figure 3:**
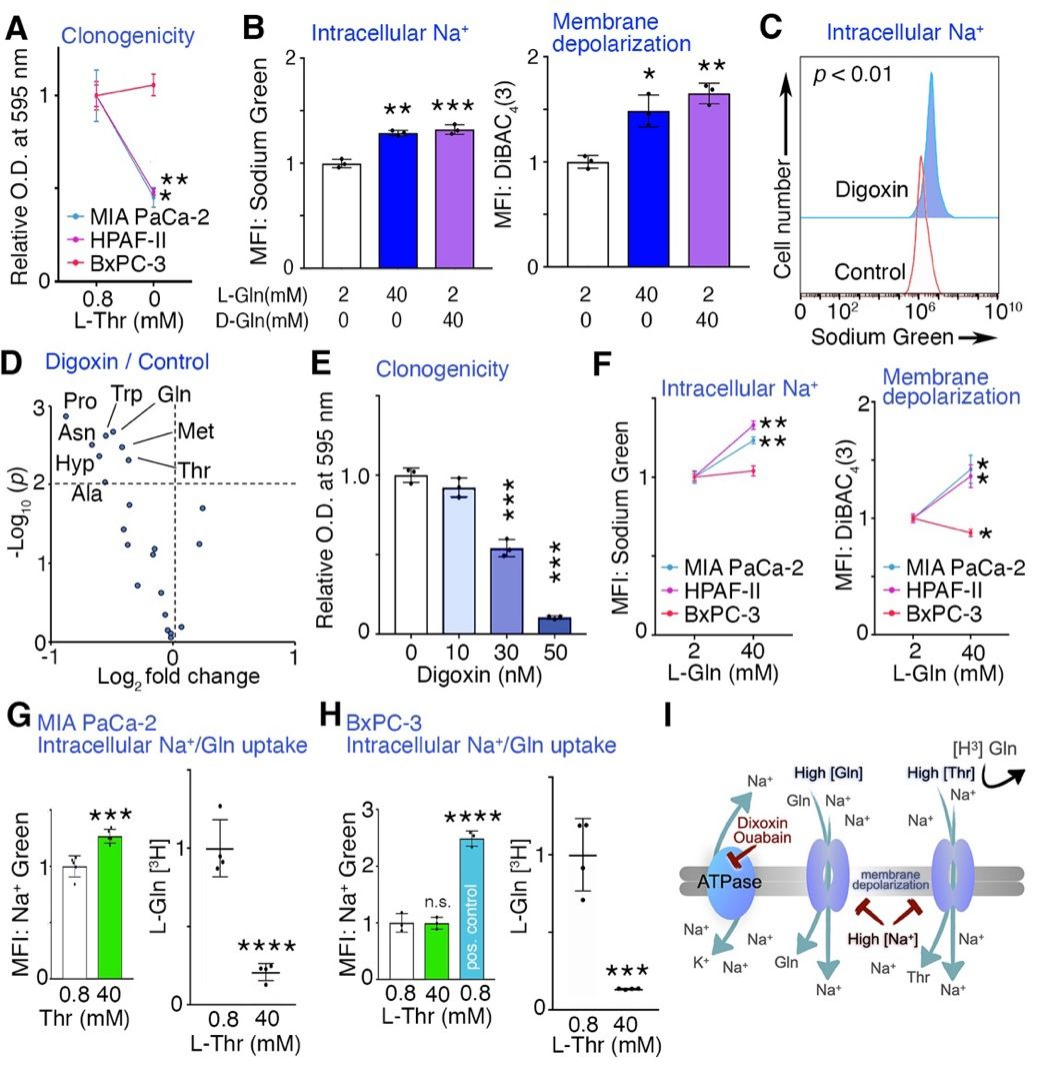
Supraphysiological amino acids modulate Na^+^ gradient and act as a competitive inhibitor for amino acid transport. (**A**) Clonogenic assays of PDAC cell lines cultured with L-Thr for 10–12 days (n = 3). (**B**) Relative intracellular sodium levels and membrane potential in MIA PaCa-2 cells exposed to indicated doses of L-Gln and/or D-Gln for 48 hrs. Mean fluorescent intensity (MFI) of Sodium Green and DiBAC_4_(3) were measured by flow cytometric analysis (n = 3). (**C**) Intracellular sodium levels in MIA PaCa-2 cells cultured with vehicle (control) or 2 μM digoxin. A representative histogram of Sodium Green uptake is shown (n = 3). (**D**) Volcano plot of differentially enriched amino acids in MIA PaCa-2 cells incubated with vehicle (control) or digoxin shown (n = 3). (**E**) Clonogenic assays of MIA PaCa-2 cells cultured with indicated concentrations of digoxin for 11 days (n = 3). (**F**) Relative intracellular sodium levels and membrane potential in PDAC cell lines cultured in indicated L-Gln concentrations (n = 3). (**G, H**) Relative intracellular sodium levels and radiolabeled L-Gln uptake in MIA PaCa-2 and BxPC-3 cells in the presence of indicated concentrations of L-Thr (n = 4). Ouabain (10 μM) was used as a positive control for the Na^+^ uptake experiment. (I) High concentrations of L-Gln or L-Thr could induce intracellular Na^+^ accumulation, membrane depolarization, and competitive inhibition for nutrient transporters, contributing to amino acid deprivation. Values are mean ± SEM. *P < 0.05, **P < 0.01, ***P < 0.001, ****P < 0.0001.

**Figure 4:**
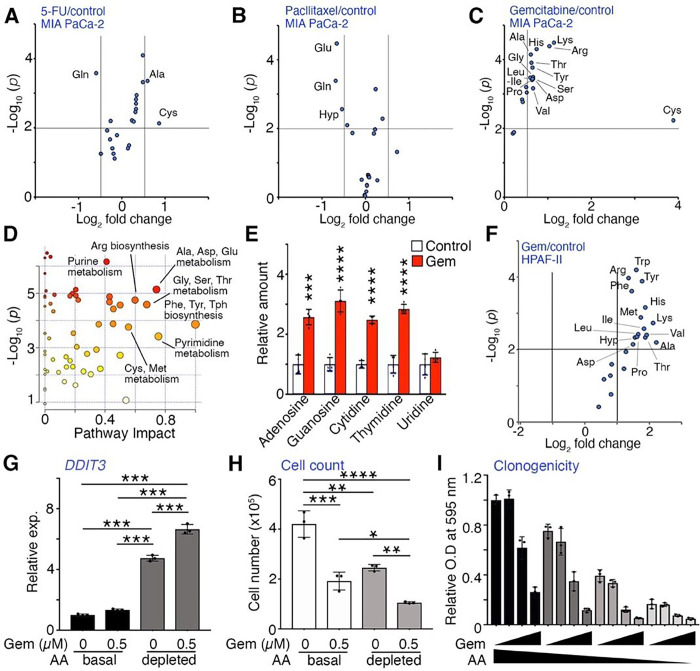
Gemcitabine-mediated reprogramming of amino acid metabolism confers vulnerabilities in PDAC cells. (**A-C**) Volcano plot of differentially enriched amino acids in response to the indicated treatments in MIA PaCa-2 cells. Metabolites with *P* < 0.01 and IFCI > 1.5 compared to control are shown (n = 3–4). (**D**) Pathway impact analysis of metabolites from MIA PaCa-2 cells treated with gemcitabine (0.5 μM for 48 hrs) compared to control. Differentially enriched pathways are indicated by color and circle size based on p-values and impact score (n = 4). (**E**) Intracellular nucleoside pools in gemcitabine (Gem)-treated MIA PaCa-2 cells relative to control (n = 4). (**F**) Volcano plot of differentially enriched amino acid levels in HPAF-II cells cultured with control or Gem (0.5 μM for 48 hrs). Metabolites with *P* < 0.01 and IFCI > 2 compared to control are indicated (n = 3). (**G, H**) *DDIT3* expression and live cell number in MIA PaCa-2 cells cultured with Gem (0 or 0.5 μM) and amino acid depleted media (AA, 1/8 of basal media) for two days (n = 3). (**I**) Clonogenic assays of MIA PaCa-2 cells cultured with gemcitabine (0, 1, 3, and 5 nM) and AA (dilutions 1, 1/4, 1/10, and 1/20) for 11 days (n = 3). Values are mean ± SEM; *P < 0.05, **P < 0.01, ***P < 0.001, ****P < 0.0001.

Several possible mechanisms were tested to explain the observed metabolic changes induced by SPG. As the media was maintained at pH 7.4 regardless of L-Gln concentration, pH was not considered a factor in the observations. Considering an amino acid salvage mechanism in RAS-transformed cells, we examined the effects of SPG^L^ on macropinocytosis measured by the uptake of high-molecular-mass FITC-dextran [10]. Consistent with past studies, lowering L-Gln concentration (< 1 mM) induced macropinocytosis in MIA PaCa-2 cells and interestingly as did SPG^L^, potentially in response to amino acid depletion (Supplementary Fig. S3A)[9, 10]. Next, considering that SPG^L^ prominently downregulated intracellular L-threonine (L-Thr) levels in three different human PDAC cell lines (Fig. 2D, E), we tested the impact of L-Thr deprivation on PDAC cells. We found that L-Thr deprivation suppressed cell proliferation as well as clonogenicity in MIA PaCa-2 and HPAF-II cells as SPG^L^ did, while BxPC-3 cells were not affected by L-Thr depletion (Fig. 3A, Supplementary Fig. S3B). Since L-Thr is an essential amino acid requiring uptake, we hypothesized that SPG decreased intracellular amino acid levels by inhibiting its uptake by some mechanisms. First, we reasoned that SPG may change sodium ion (Na^+^) gradient that regulates the functions of Na^+^-dependent transporters for both Thr and Gln uptake, as observed in oocytes by others [23, 29]. In fact, after 6 hrs of supraphysiological L-Gln treatment, there was an increase in intracellular Na^+^ levels and plasma membrane depolarization, measured by Na^+^-sensitive fluorescent probe Sodium Green and DiBAC_4_(3), respectively (Supplementary Fig. S3C, D). Of note, supraphysiological D-Gln has similar effects in MIA PaCa-2 cells (Fig. 3B). Na^+^/K^+^-ATPase activity can help establish the Na^+^ gradient, and its inhibition by digoxin and ouabain (Na^+^/K^+^-ATPase inhibitors) predictably increased intracellular Na^+^ levels (Fig. 3C and Supplementary Fig. S3E). This paralleled broad reduction of intracellular amino acid levels and clonogenicity in digoxin treated MIA PaCa-2 cells (Fig. 3D, E). The treatment of MIA PaCa-2 cells with ouabain similarly suppressed cell count and colony formation in a dose-dependent manner (Supplementary Fig. S3F, G). While SPG^L^ elevated intracellular Na^+^ levels and mediated plasma membrane depolarization in MIA PaCa-2 and HPAF-II cells, these changes were not observed in BxPC-3 (Fig. 4F). Unexpectedly, the UN-KPC-960 and HCT116 cells that showed sensitivity to SPG in cell growth did not demonstrate intracellular Na^+^ accumulation (Supplementary Fig. S3H). These incongruous observations suggested the modulation of intracellular Na^+^ levels or membrane potential may not entirely explain the underlying mechanism contributing to the observed effects of SPG.

To determine if supraphysiological concentrations of amino acids can inhibit amino acid uptake without affecting intracellular Na^+^ levels, other amino acids can act similarly to Gln, we examined the effects of supraphysiological concentrations of L-Thr on amino acid uptake in PDAC cells. We chose L-Thr since it is also a substrate of the glutamine transporter, ASCT2. Similar to SPG treatment, supraphysiological L-Thr potentiated Na^+^ uptake in MIA-PaCa-2 cells, but had no effect in BxPC-3 cells where ouabain treatment demonstrated an elevated intracellular Na^+^ accumulation as a positive control (Fig. 3G, H). Of note, when either MIA-PaCa-2 or BxPC-3 cells were incubated with supraphysiological L-Thr, the uptake of L-Gln-[^3^H] was significantly decreased. These data suggested that despite the differential responses of Na^+^ accumulation/membrane potential in the diverse PDAC cell lines, supraphysiological concentrations of amino acids, such as L-Gln and L-Thr could paradoxically decrease intracellular amino acid levels by competitive inhibition of their transporters (Fig. 3I). Interestingly, supraphysiological L-Thr had similar growth inhibitory effects in MIA PaCa-2 cells as SPG (Supplementary Fig. S3I), supporting the idea that this strategy could be applicable to other amino acids.

### Gemcitabine-mediated intracellular amino acid enrichment confers vulnerability in PDAC cells.

Next, we focused on the metabolic changes caused by chemotherapy that could be a targetable vulnerability in PDAC patients. Metabolomic analysis demonstrated a broad enrichment of amino acids with gemcitabine treatment, while 5-fluorouracil (5-FU) and paclitaxel treatment had few differentially enriched amino acids (Fig. 4A–C). Of note, we used an IC_50_ dose of each drugs for metabolomics, such as 0.5 μM for gemcitabine that caused an increase in the sub-G1 apoptotic and S phase cell cycle arrest (Supplementary Fig. S4A, B). The apparent uracil accumulation was assumed to be caused by the deamination of gemcitabine followed by further degradation to uracil (Supplementary Fig. S4C). Pathway analysis revealed ‘phenylalanine, tyrosine, and tryptophan biosynthesis,’ ‘pyrimidine metabolism, alanine, aspartate, and glutamate metabolism,’ ‘glycine, serine and threonine metabolism, arginine biosynthesis,’ as the most significantly altered networks (Pathway Impact > 0.6; Fig. 4D). Moreover, we found that gemcitabine treatment resulted in a significant enrichment of nucleosides (Fig. 4E), while nucleotide pools were unevenly present (Supplementary Fig. S4D). In addition to the expected changes in nucleic acid metabolism by gemcitabine, we found an accumulation of 20 amino acids replicated in HPAF-II cells in support of a broader metabolic impact of gemcitabine treatment (Fig. 4F). The intracellular enrichment of essential amino acids was associated with significant upregulation of several amino acid transporters by gemcitabine treatment in MIA PaCa-2 cells (Supplementary Table. S1). PDAC patients are also reported to have elevated expression of Gln transporters, *SLC1A5, SLC3A2* and *SLC7A5* [30–32].

As cysteine was the most significantly enriched amino acid by gemcitabine treatment in MIA PaCa-2 cells, its downstream metabolites, glutathione (GSH, γ-glutamyl-cysteinyl-glycine) and glutathione disulfide (GSSG) were also found to be elevated, probably associated with amelioration of reactive oxygen species (ROS) (Supplementary Fig. S4E). Accordingly, the cognate cystine/cysteine transporter, solute carrier family 1 member 4 (*SLC1A4*, also known as ASCT1) was significantly upregulated by gemcitabine, but the expression of another cysteine transporter, *SLC7A11* (also known as xCT) was not increased in MIA PaCa-2 and BxPC-3 cells (Fig. S4F). The knockdown of either xCT or ASCT1 by siRNA elevated intracellular ROS levels under basal conditions with further elevation in the context of gemcitabine, paralleling decrease in MIA-PaCa-2 cell proliferation (Supplementary Fig. S4G, H). However, in BxPC-3 cells, the knockdown of either xCT or ASCT1 did not affect cell growth in the context of gemcitabine. Although inhibiting individual amino acid transporters had differential effects on PDAC lines, a general deficiency of amino acid in the media (1/8 of basal media) predictably induced ER stress, as indicated by the upregulation of C/EBP homologous protein (CHOP, encoded by the DNA damage-inducible transcript 3 *DDIT3*) gene and decreased cell proliferation in both MIA PaCa-2 and BxPC-3 (Fig. 4G, H; Supplementary Fig. S4I). The combination of amino acid starvation and gemcitabine treatment had an additive effect in reducing colony formation compared to gemcitabine alone in a dose dependent manner, based on the Chou-Talalay’s method of synergy determination (combination index, CI = 1; Fig. 4I) [33]. Considering the observed amino acid enrichment by gemcitabine, it was not surprising that gemcitabine sensitization in PDAC cells was achieved by broad amino acid deprivation.

### Supraphysiological glutamine sensitizes PDAC tumors to gemcitabine

**Figure 5:**
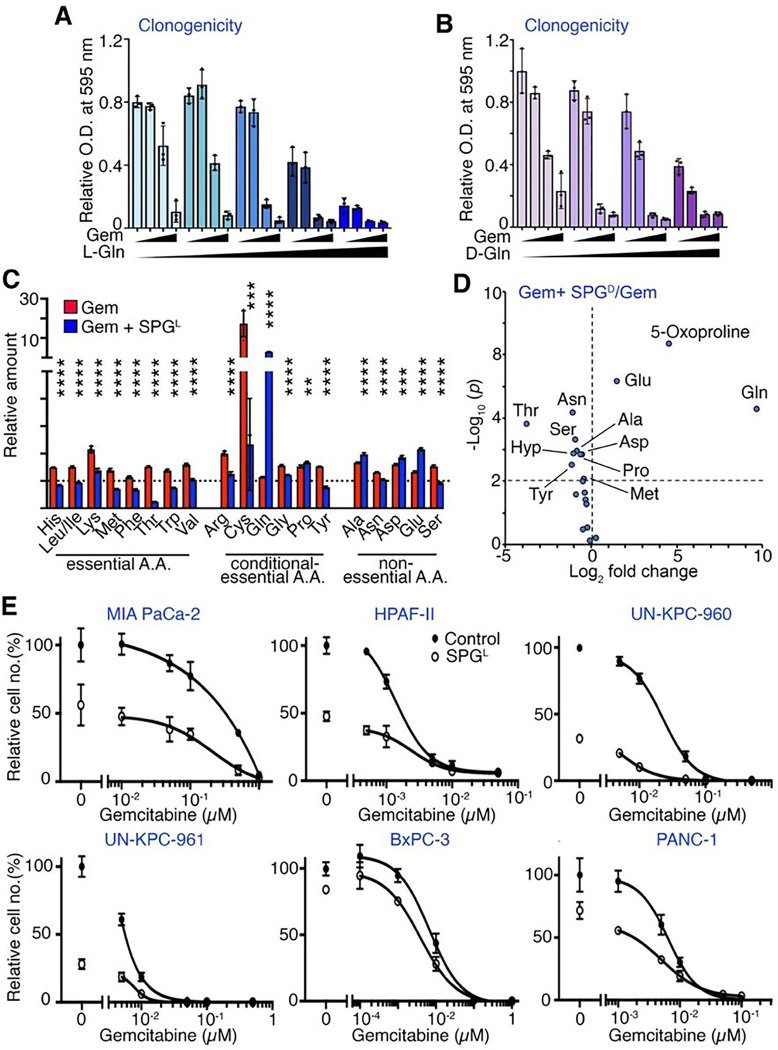
Supraphysiological Gln antagonizes Gem-induced metabolic reprogramming and sensitizes PDAC cells to Gem. (**A, B**) Clonogenic assays of MIA PaCa-2 cells were performed with gemcitabine (Gem, 0, 1, 3 and 5 nM) and L-Gln (2, 10, 20, 30 and 40 mM) or D-Gln (0, 2, 20, and 40 mM, in the presence of 0.5 mM L-Gln) (n = 3). (**C**) Intracellular amino acid levels in MIA PaCa-2 cells cultured with 0.5 μM gemcitabine and SPG L or gemcitabine alone. The values were normalized to cells treated with 2 mM L-Gln (n = 4). (**D**) Volcano plot of differentially enriched amino acids in MIA PaCa-2 cells following SPG^D^/Gem or Gem treatment. Metabolites with *P* < 0.01 are indicated (n = 3). (**E**) PDAC cell lines were cultured in 2 mM (control) or 40 mM L-Gln (SPG^L^) with different concentrations of gemcitabine for four days for IC_50_ determination (n = 3). Values are mean ± SEM; **P < 0.01, ***P < 0.001, ****P < 0.0001.

We rationalized that increased amino acid pools in gemcitabine-treated PDAC cells could be targeted by SPG based on its ability to inhibit amino acid uptake. Clonogenic assays demonstrated higher concentrations of L-Gln or D-Gln significantly reduced the concentration of gemcitabine required to yield similar colony numbers as seen under basal media for MIA PaCa-2 cells with higher doses of gemcitabine (Fig. 5A, B). Chou-Talalay’s Combination Index calculations indicated an additive effect for either supraphysiological L-Gln or D-Gln and gemcitabine. Incidentally, SPG^L^ similarly sensitized MIA PaCa-2 cells to 5-FU and paclitaxel in colony formation assays despite their lack of appreciable amino acid enrichment as a single agent (Supplementary Fig. S5). In determining the metabolic changes caused by the combined treatment of SPG^L^ with IC_50_ dose of gemcitabine, we found four distinct metabolic responses, depicted by an unsupervised hierarchical cluster analysis and PCA analysis, differentiated the four treatment conditions (Supplementary Fig. S6A, B). Importantly, supraphysiological L-Gln and D-Gln limited gemcitabine-induced accumulation of intracellular amino acids inclusive of all essential amino acids (Fig. 5C, D). Similar decrease in intracellular amino acid pools was observed in combined SPG^L^ and gemcitabine-treated HPAF-II cell (Supplementary Fig. S6C). Accordingly, we found SPG^L^ resulted in a lower gemcitabine IC_50_ in MIA PaCa-2, HPAF-II, UN-KPC-960 and UN-KPC-961 cells compared to that in basal media, with a lesser effect on BxPC-3 and PANC-1 cells (Fig. 5E). SPG reversed gemcitabine-mediated amino acid enrichment and caused upregulation of *DDIT3* and intracellular ROS levels (Supplementary Fig. S6D, E). Parallel RNA sequencing analysis demonstrated that SPG inhibited gemcitabine-stimulated expression of *SLC2A3* and *SLC2A4* that encode the glucose transporters GLUT3 and GLUT4, in line with observed glucose and lactate enrichment (Supplementary Fig. S6F, G). Consistent with the elevated S-phase population identified by cell cycle distribution analysis, *H2BC12* and *H2AC6* expression were significantly elevated by SPG^L^ under gemcitabine treatment, compared to gemcitabine alone (Supplementary Fig. S7A, B). Furthermore, several interferon response-related gene signatures obtained from GSEA appeared elevated under combination treatment (Supplementary Fig. S7C). Together, these data suggest SPG^L^ antagonized pivotal metabolic and cell cycle changes required for PDAC survival under gemcitabine treatment.

**Figure 6:**
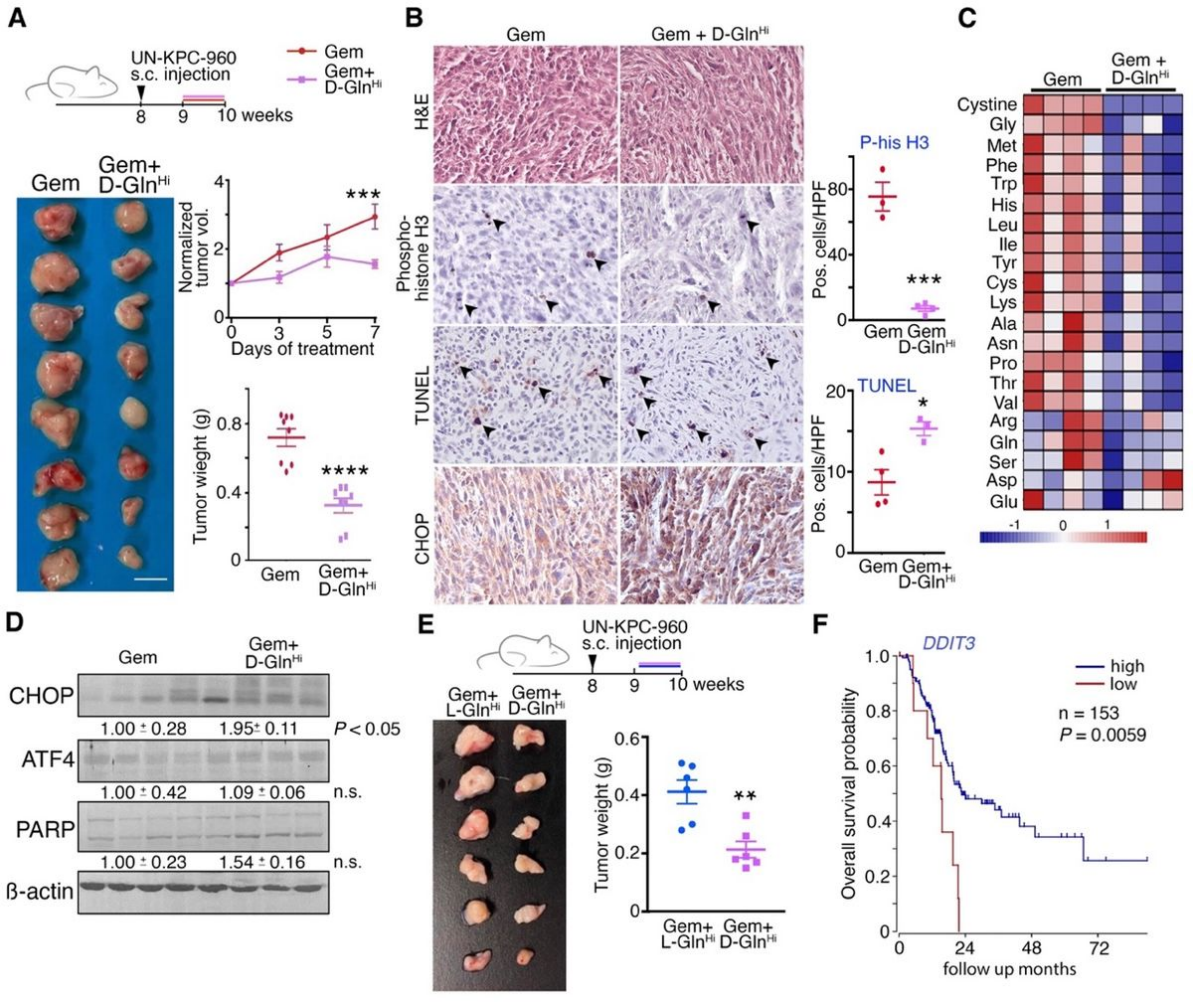
The effects of Gln supplementation on subcutaneous PDAC allografts. (**A**) Schematic of the study design represents UN-KPC-960 subcutaneous allografting of male athymic nude mice. One week after tumor introduction, the mice were challenged with gemcitabine (Gem, 50 mg/kg, i.p., twice a week) alone or in combination with D-Gln (30 mg/kg, s.c., daily). Tumor volumes were measured every 2–3 days and tumor weights were measured at the endpoint (n = 8). (**B**) Tumor tissues were subjected to hematoxylin and eosin (H&E) and immunohistochemical (IHC) staining. Positive staining of phosphorylated histone H3 and TUNEL was quantitated per high powered field (HPF, n > 3). (**C**) Metabolomic analysis of differentially enriched amino acids in the allografts were measured following treatment of mice with Gem alone or with D-Gln was depicted in a heatmap (n = 4). (**D**) Western blot analysis of tumor tissues for the indicated proteins with significance of the differences between the groups indicated right of the lane. (**E**) Schematic of the study design represents UN-KPC-960 subcutaneous allografting of male athymic nude mice. One week after tumor introduction, the mice were given Gem (50 mg/kg, i.p., twice a week) in the presence of L-Gln or D-Gln (30 mg/kg, s.c., daily). Tumor weights at endpoint were indicated (n = 6). (**F**) Kaplan-Meier curves represent overall survival probability of pancreatic cancer patients according to DDIT3 expression considered as a dichotomous variable in the TCGA mixed pancreatic adenocarcinoma dataset. Values are mean ± SEM; *P < 0.05, **P < 0.01, ***P < 0.001, ****P < 0.0001.

To test the near-term therapeutic potential of SPG, we orthotopically grafted UN-KPC-960 into syngeneic mice for treatment with gemcitabine alone (50 mg/kg, i.p. twice weekly) or in combination with L-Gln (250 mg/kg, orally twice daily). After allowing the tumors to expand for one week, the mice were subjected to 8-weeks of treatment. The harvested PDAC tumors exhibited limited mitotic index (*P* < 0.05) and elevated terminal deoxynucleotidyl transferase dUTP nick end labeling (TUNEL; *P* < 0.001) by combination therapy, compared to gemcitabine alone (n = 8; Supplementary Fig. S8). Next, to improve longitudinal assessment of heterogenous tumor growth rates among the mice and to achieve supraphysiological Gln concentrations in tumors, subcutaneously grafted UN-KPC-960 tumors were treated at approximately 1 cm^3^ with gemcitabine in the presence or absence of supraphysiological D-Gln (30 mg/kg/day, s.c. daily; Fig. 6A). Combination therapy significantly suppressed tumor growth compared to gemcitabine alone (volume: *P* < 0.001, weight: *P* < 0.0001). The tumors from mice treated with combination therapy had greater apoptotic or necrotic regions, determined by TUNEL (*P* < 0.05), accompanied by limited mitosis, determined by phosphorylated-histone H3 immuno-localization (*P* < 0.001; Fig. 6B). Similar to in vitro data, the tumor tissues demonstrated an elevated CHOP expression when mice were given gemcitabine/D-Gln combination, supported by the dramatic reduction in gemcitabine-induced amino acid enrichment (*P* < 0.0001; Fig. 6C). Western blotting of the tumors from combination therapy treated mice demonstrated elevated ER stress by the significant induction of CHOP (*P* < 0.05), indicating the induction of nutrient deprivation (Fig. 6D). To compare the effects of the glutamine enantiomers on gemcitabine sensitivity, UN-KPC-960 tumors were allografted and treated for 1 week with either supraphysiological L-Gln or D-Gln in the presence of gemcitabine (Fig. 6E). The resulting tumors following gemcitabine/D-Gln combination were significantly smaller than those treated with the gemcitabine/L-Gln combination (*P* < 0.01). Importantly, there was no evidence in altered liver function based on standard enzyme expression (LDH, ALP, ALT, and AST) or body weight among the treatment groups (Supplementary Fig. S9). While there are many mechanisms for the induction of *DDIT3* (CHOP), a demonstrated indicator of overall survival of PDAC patients (*P* = 0.0059, n = 153; Fig. 6F), cancer-specific nutrient deprivation is a clinically viable means of achieving such expression. Taken together, our data suggested that supraphysiological D-Gln could work as a gemcitabine sensitizer via amino acid deprivation and inhibition of anaplerosis.

## Discussion

Antagonism of Gln metabolism has been a foundational approach for targeting PDAC metabolism by either blocking glutamine transporters or glutaminase [3, 34, 35]. However, these efforts do not provide durable benefit due to multiple adaptive mechanisms [7]. SPG suppressed the proliferation of diverse cancer cell types limiting amino acid, nucleotide pools, and promoting cell cycle S phase arrest, all in the context of elevated mitochondrial respiration revealing a metabolic imbalance (Fig. 1, 2). While not entirely surprising, SPG^D^ deprived the cells of amino acids uptake as well as the ATP derived from oxidative phosphorylation that may give SPG^D^ a superior anti-tumor capacity. Further studies including the isotope-labeled metabolic tracing studies with both L-Gln and D-Gln should be performed to determine the fates of SPG in cancer cells. The capacity for SPG to broadly deplete amino acids brings into question the apparent tumor-selectivity of the treatment strategy. In addition to the fact that there are greater amino acid needs in cancer cells, cancer cells are known to be especially sensitive to membrane depolarization and have elevated expression of Gln transporters [36, 37], ideal for SPG treatment. Modulation of intracellular Na^+^ concentrations using Na^+^/K^+^-ATPase blockers caused amino acid depletion to serve as a proof of principle for the inhibition of Na^+^-dependent amino acid transport, while it may have some other mechanisms (Fig. 3) [26]. Since threonine was one of the most depleted amino acids by SPG, an essential amino acids that can be obtained only through an uptake, it suggest that SPG may inhibit amino acid uptake in PDAC cells. We tested the effects of threonine deprivation on cancer cell growth to find its growth inhibitory effects in PDAC cells. The depletion of L-Thr could have a similar consequence as the depletion of L-Gln in proliferative cancer cells [38]. We also tested the effects of high concentrations of L-Thr and discovered a competitive inhibition of amino acid transport limiting the uptake of radiolabeled-L-Gln. Considering the redundancies in L-Gln and L-Thr transporters, often having multiple amino acid substrates, our data would suggest that high doses of multiple amino acids may yield similar results in cancer cells.

While the reported enteric benefits of SPG are fortuitous in context of its complementation of the metabolic reprogramming induced by chemotherapy [39], our metabolic profiling of the gemcitabine-treated PDAC cells revealed enrichment of nucleic acids and amino acids, not observed with 5-FU or paclitaxel, exposing vulnerability to SPG-induced metabolic reprograming (Fig. 4). Prior studies have profiled PDAC cell lines following long-term gemcitabine exposure and identified some similar metabolic changes [40, 41]. Consistent with this result, we observed a significant decrease in essential amino acids in the blood of PDAC patients receiving gemcitabine-based chemotherapy (unpublished). The reversal of the gemcitabine-induced amino acid enrichment by SPG elevated intracellular ROS, contributing to elevated cell death (Fig. 5). SPG was found to diminish gemcitabine-induced amino acid enrichment and promote ER stress in multiple PDAC cell lines and the tumor allografts. Proline, serine, and glycine deprivation-based induction of endoplasmic reticulum stress is reported to complement chemotherapy in PDAC models [42, 43]. However, although both low Gln concentrations and SPG induce ER stress, SPG doesn’t have similar effects on some Gln deprivation-responsive-genes (Supplementary Fig. S1). Both Gln enantiomers were found to decrease nucleotides such as UTP and CTP that may enhance the effects of nucleoside analog gemcitabine. The observed ability for SPG to also sensitize MIA PaCa-2 cells to 5-FU and paclitaxel was likely due to the inability to replicate/repair DNA resulting from the induced deficiency of nucleic acids, based on the depletion of the precursor phospho-ribose-pyrophosphate (PRPP) and decreased glucose uptake for pentose phosphate pathway (Supplementary Fig. S5 and S6).

The Gln concentrations used in culture cannot be achieved in serum. However, the dose of Gln (30 mg/kg/day) given subcutaneously in mice was much lower than that prescribed for sickle cell anemia patients (300 mg/kg/day, orally) (Fig. 6) [20]. It was sufficient to inhibit growth of established tumors by the revealed mechanism of amino acid depletion. A current phase 1 clinical trial (NCT04634539) is evaluating whether L-Gln (Endari^®^) supplementation for PDAC patients receiving gemcitabine and Nab-paclitaxel is safe with preliminary evidence of anti-tumor activity as a secondary outcome measure [44]. Although repurposing of FDA approved L-Gln may allow for expeditious translation, the development of a clinically viable D-Gln formulation would best reflect the benefits of the gemcitabine-induced synthetic lethality observed in the preclinical studies here. We observed limited alteration of Gln levels in the tumors of mice given high dose D-Gln potentially due to harvesting several hours after the last administration. This revealed that the general amino acid depletion and associated ER stress can persist even after possible D-Gln efflux or hydrolysis, not explored here [45]. Further, the observed amino acid depletion in the context of elevated macropinocytosis was curious. We wonder if the observation may be because energy intensive lysosomal activity following protein uptake may have been impaired. Clinically, gemcitabine is rarely given as a single agent to PDAC subjects, rather often combined with Nab-paclitaxel. Unfortunately, the available model systems were sensitive to the combination chemotherapy, making it difficult to show further superiority of the SPG. Since there are some studies that indicate the role of D-amino acids in cancer, long term effects of its administration need to be determined [46]. The findings could be broadly applicable to other malignancies with additional chemotherapies where metabolic dependencies could be exploited by a supplementation of high-dose non-metabolizable amino acids.
